# Active schistosomiasis, severe hypereosinophilia and rapid progression of chronic endomyocardial fibrosis

**DOI:** 10.5830/CVJA-2016-030

**Published:** 2016

**Authors:** AO Mocumbi, A Damasceno, C Carrilho, C Goncalves

**Affiliations:** Instituto Nacional de Saúde, Maputo, Mozambique; Eduardo Mondlane University, Maputo, Mozambique; Eduardo Mondlane University, Maputo, Mozambique; Maputo Central Hospital, Mozambique; Eduardo Mondlane University, Maputo, Mozambique; Maputo Central Hospital, Mozambique; Maputo Central Hospital, Mozambique

**Keywords:** endomyocardial fibrosis, schistosomiasis, pathogenesis, management

## Abstract

Endomyocardial fibrosis (EMF) is a neglected restrictive cardiomyopathy of unknown aetiology and unclear natural history, which causes premature deaths in endemic areas. We present the case of a 13-year-old boy from a highly endemic area, presenting with concurrent signs of chronic EMF and severe hypereosinophilia associated with active schistosomal cystitis. We discuss the possible role of this parasitic infection in determining the progression of EMF in endemic areas for both conditions.

## Abstract

Endomyocardial fibrosis (EMF) is a poorly understood restrictive cardiomyopathy that affects mainly children and adolescents in endemic areas of Africa, Asia and Latin America.^1^ The suggested pathogenesis is that of succession of necrosis, thrombosis and fibrosis, but this has been difficult to prove because most patients are seen in late stages of the disease. We describe a case of EMF with severe fibrosis associated with active schistosomiasis, hypereosinophilia and a fatal outcome.

## Case report

A 13-year-old boy of black ethnicity from an endemic zone of EMF was referred to hospital in congestive heart failure.A 13-year-old boy of black ethnicity from an endemic zone of EMF was referred to hospital in congestive heart failure.

He reported a three-month history of progressive exertional dyspnoea without orthopnoea or paroxysmal nocturnal dyspnoea, as well as central, crushing and constant chest pain, which was exacerbated by exercise and alleviated on rest. He also complained of progressive painless abdominal distension, but denied having palpitations, wheeze, cough, night sweats, fever, or any gastrointestinal or urinary symptoms. His past medical history was uneventful, and he was not on medication prior to his first admission, two weeks before he was transferred.

On examination he was alert, apyretic, had no neurological signs of disease or disorientation and presented a good general status. His heart rate was 108 beats/minute with a regular rhythm, blood pressure was 90/60 mmHg, and respiratory rate was 16 breaths/minute.

Cardiac examination revealed raised jugular venous pressure, a visibly pulsating, palpable, non-displaced apex beat, and a mild holosystolic murmur on auscultation. Besides a bilateral inspiratory wheeze, the respiratory examination was unremarkable.

The abdomen was soft, non-tender and mildly distended, with a 3-cm hepatomegaly and no other organomegaly. He was not jaundiced and there was no shifting dullness on abdominal examination.

Blood examinations for malaria, human immunodeficiency virus, recent streptococcal infection, rheumatoid factor and syphilis were all negative. Erythrosedimentation rate was raised at 55 mm/h. White blood cell count was normal with marked eosinophilia. Stool examination for helminths was negative.

The chest X-ray showed prominence of the pulmonary artery. The ECG revealed sinus rhythm, signs of right ventricular overload and non-specific repolarisation abnormalities.

On transthoracic echocardiography, the right ventricular cavity was reduced and areas of endocardial thickening suggested fibrosis; the overall right ventricular function was preserved. The right atrium was dilated and moderate tricuspid regurgitation was present, allowing estimation of systolic pulmonary pressure at 85 mmHg. No images suggesting thrombi were detected on the right side of the heart. The left ventricle had marked thickening of the mural endocardium and a homogeneous mass occupying its apical third, suggesting a thrombus that did not interfere with the mitral valve function. Left ventricular systolic and mitral valve function were preserved; mild circumferential pericardial effusion was present.

The results of rectal biopsy were inconclusive for schistosomal infection.

A diagnosis of bilateral EMF with hypereosinophilia was made and the patient was managed with daily oral furosemide 40 mg, spironolactone 25 mg, warfarin 1.25 mg, and a single dose of 400 mg albendazole. Despite improvement with resolution of dyspnoea and chest pain after 24 hours, the patient died unexpectedly on day 6 while sleeping.

**Fig. 1:**
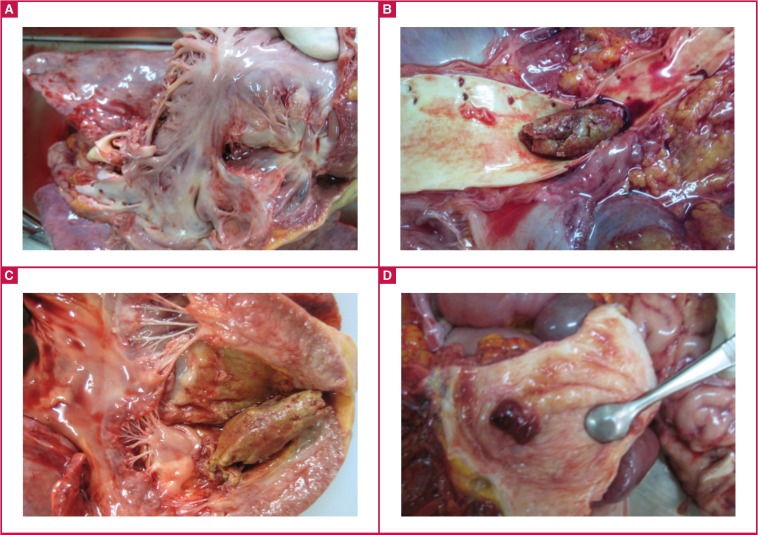
Typical features of right ventricular EMF include fibrosis and retraction (A). A large embolus that could have been seated at the left ventricular apex (B) is seen at the bifurcation of the abdominal aorta. Macroscopic evaluation also revealed extensive left ventricular endocardial fibrosis (C) and bilharzia polypoid cystitis (D).

Autopsy confirmed right ventricular diffuse endocardial fibrous thickening with amputation of the apex Fig. 1A, aneurysmal dilatation of the right atrium, and extensive endocardial fibrosis of the left ventricle. The bifurcation of the aorta was filled with a large embolus Fig. 1B that could have been seated at the left ventricular apex Fig. 1C. The bladder revealed active polypoid bilharzial cystitis Fig. 1D.

**Fig. 2:**
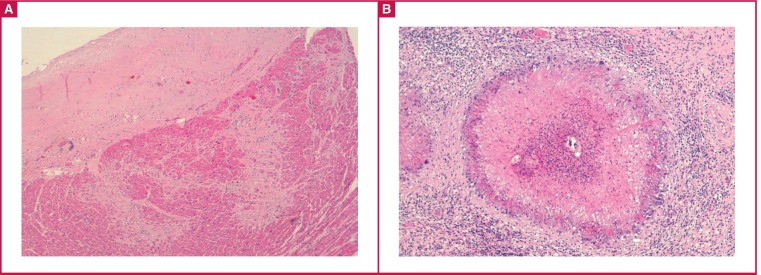
Histological features of EMF include endocardial thickening by fibrosis, with strands of fibrous tissue penetrating the inner myocardium (A). Eosinophilic granulomas centred by viable Schistosoma eggs were found in the bladder (B).

On microscopy, typical endocardial fibrous thickening Fig. 2A and eosinophilic granulomas centred by viable Schistossoma eggs were found Fig. 2B. Additional features were chronic passive congestion of the liver, spleen and lung, as well as hepatic periportal fibrosis with the presence of eosinophilic granulomas.

## Discussion

This patient, coming originally from a known endemic region for EMF, had bilateral disease. He had concurrent signs of severe endocardial fibrosis, marked tissue hyper-eosinophilia and active Schistosoma haematobium granuloma in the bladder. He therefore presented with signs of both chronic EMF and active schistosomal infestation, as defined by the presence of viable eggs and active granuloma.

The patient had been relatively asymptomatic until three months prior to admission, in marked contrast with the severity of the echocardiographic and pathological features. Discrepancy between echocardiographic and clinical findings is not uncommon,^2^ and recent schistosomiasis may have contributed to aggravation of a stable chronic EMF.

Although emergency surgery had been considered when the child was admitted, it was not performed due to the presence of extensive endocardial fibrosis with severe ventricular cavity amputation, pulmonary hypertension and electrocardiographic signs of myocardial ischaemia, all predictors of a bad prognosis. Since antithrombotic therapy was unavailable, the child was treated with warfarin only. Sudden death occurred probably due to ventricular arrhythmia that may have been determined by dislodgment of the large apical left ventricular thrombus and embolisation to the aortic bifurcation.

Loffler’s syndrome is used as a model to explain some clinical– pathological features of EMF,^3^ but eosinophilic myocarditis is rarely proven in these patients. Endomyocardial biopsy is rarely performed due to lack of expertise and non-existence of adequate facilities for catheterisation in endemic areas, as well as the presence of advanced disease and intracavitary thrombi, as was the case in our patient.

EMF can be associated with parasitic infestations and their attendant eosinophilia. An association with Schistosoma mansoni, haematobium and intercalatum has been reported.^4^,^5^,^6^ In the our case, Schistosoma infection may have been the trigger for clinical aggravation due to the superimposition of Loeffler’s syndrome on chronic fibrotic EMF. Although we found no calcification or other signs of chronic schistosomiasis, we cannot exclude previous episodes.

## Conclusion

Our findings support the hypothesis of EMF being a progressive disease that may be linked to repetitive inflammatory insults, which may correspond with successive episodes of blood and endomyocardial hypereosinophilia triggered by parasitic infestation or other factors. They also suggest the need to explore new management approaches, including prevention of recurrences in patients with chronic, established disease. This should probably involve strict control of endemic parasitic infections, as well as the use of anti-inflammatory drugs and anticoagulants, mimicking the current standard of care in Loeffler’s syndrome. The EMF diagnosis and scoring system previously used in community screening, considering only the severity and distribution of structural lesions,^2^ could probably be improved to include inflammation, cardiac and coagulation biomarkers, thereby allowing its use for risk stratification and tailored management of this neglected cardiomyopathy.
